# Supplementary material for: Predictors of rapid eye movement sleep behavior disorder in patients with Parkinson’s disease based on random forest and decision tree
Source: PLoS One. 2022 Jun 16;17(6):e0269392. doi: 10.1371/journal.pone.0269392 (PMC9202951; doi:10.1371/journal.pone.0269392)
Supplement: Attachment — Submitted filename: Response to Reviewers.doc [file pone.0269392.s001.doc]

Dear Editors and Reviewers,

On behalf of all co-authors, we appreciate you very much for the positive and constructive feedback on our manuscript entitled “Predictors of rapid eye movement sleep behavior disorder in patients with Parkinson's disease based on random forest and decision tree”. (ID**:**PONE-D-21-37123). We have studied reviewers’ comments carefully and have made revisions to the manuscript based on your comments. Below are details of how I have addressed the point raised. All of the changes have been highlight.

**Responds to the reviewer’s comments:**

1.The paper definitely needs English editing.

**Responds:** We are very sorry for the poor writing. The manuscript has been polished by the native speaker.

2.Focus should also be made on using the same abbreviations everywhere in text (for example for PD without RBD: NO P-RBD, NP-RBD, PD-NRBD, PD-nRBD; which

abbreviation should be used?)

**Responds:** We are so sorry for the informal spelling and we have checked all the abbreviations in the manuscript.

3.References should be included everywhere in text, where needed (for example the rating scales for motor and nonmotor symptoms in Materials and Methods)
**Responds:** Thank you so much for your comments, we have added related references in the manuscript.

4.In Materials and Methods it is mentioned: ‘A total of 102 patients with Parkinson's disease who were admitted to…’and in the rest of the paper 169 patients are mentioned. What do the 102 represent?

**Responds:** We are so sorry for the spelling mistake. It should be 169 patients.

5.The conclusions should be rephrased and presented more clearly in relation to the results.

**Responds:** Considering your valuable suggestion, we have rewritten the conclusion to make it more clear.
