# Supplementary material for: Predictors of rapid eye movement sleep behavior disorder in patients with Parkinson’s disease based on random forest and decision tree
Source: PLoS One. 2022 Jun 16;17(6):e0269392. doi: 10.1371/journal.pone.0269392 (PMC9202951; doi:10.1371/journal.pone.0269392)
Supplement: Attachment — Submitted filename: Response to Reviewers.doc [file pone.0269392.s003.doc]

Dear Editors and Reviewers,

On behalf of all co-authors, we appreciate you very much for the positive and constructive feedback on our manuscript entitled “Predictors of rapid eye movement sleep behavior disorder in patients with Parkinson's disease based on random forest and decision tree”. (ID**:**PONE-D-21-37123). We have studied reviewers’ comments carefully and have made revisions to the manuscript based on your comments. Below are details of how I have addressed the point raised. All of the changes have been highlight.

**Responds to the editors’ comments:**

The reference list has been checked to ensure the complete and correct.

**Responds to the reviewer’s comments:**

Thank you so much for pointing the inappropriate words in the manuscript. We have revised them.
